# A Generation at Risk: The Impacts of Lebanon's Escalating Humanitarian Crisis on Children

**DOI:** 10.3389/fpubh.2021.704678

**Published:** 2021-08-16

**Authors:** Marian Abouzeid, Dana A. Halwani, Ali H. Mokdad, Rima R. Habib

**Affiliations:** ^1^Alfred Deakin Institute for Citizenship and Globalisation, Deakin University, Burwood, VIC, Australia; ^2^Faculty of Health Sciences, American University of Beirut, Beirut, Lebanon; ^3^Institute for Health Metrics and Evaluation, University of Washington, Seattle, WA, United States

**Keywords:** humanitarian crisis, children, youth, Lebanon, child well-being, child health, political determinants of health

## Abstract

Lebanon is in the midst of a rapidly escalating, unprecedented humanitarian crisis that is plunging the country deep into poverty and threatens population well-being, economic development, social welfare and national and regional stability. The dire situation is due to the compounding effects of the August 2020 Beirut blast, massive economic collapse and the COVID-19 pandemic, in a setting of longstanding entrenched political corruption and a dysfunctional, mismanaged crisis response by the state. This current emergency occurs on the background of a turbulent history and complex regional geopolitical context – including the Syrian refugee crisis, the ongoing influence of foreign actors and their local proxies, the United-States-imposed sanctions, endemic corruption, a culture of nepotism and entitlement among the political dynasties, dysfunctional power-sharing and deep-seated sectarian divides. With over half the population now living in poverty, a generation of children are among those at risk. This Perspective provides a brief overview of Lebanon's current complex humanitarian crisis, discusses the impacts of the evolving situation on youth and proposes a suite of recommendations to mitigate the effects.

## Introduction

Lebanon is in the midst of an escalating humanitarian emergency arising from the compounding effects of three concurrent crises: massive economic collapse, the August 4 ammonium nitrate blast at the Port of Beirut and the COVID-19 pandemic. These crises come on the background of a complex local and regional geopolitical context, including a confessional political establishment characterised by endemic corruption and generating entrenched structural inequalities; a long history of foreign occupations and recurrent incursions that have had lasting impacts on critical infrastructures and stability; ongoing foreign influences that have rendered Lebanon a proxy battleground and continue to destabilise; the issue of over 540,000 Palestinian refugees in Lebanon (1), highly vulnerable and denied the most basic of rights, continuing to fuel social tensions; and the effects of a decade-long conflict in neighbouring Syria, which has seen the influx of over one million refugees into Lebanon, straining services and infrastructures and exacerbating an already tense social environment in the country. Together these crises have precipitated political turmoil and a dire, unprecedented humanitarian emergency that is plunging the country deep into poverty and threatens population well-being, economic development, social welfare and national and regional security. With more than half the population now living in poverty (2), a generation of children are among those at risk. UNICEF has warned that children are the worst hit, with every aspect of their lives including health and safety at risk and their futures at stake (3). Urging immediate action, Save the Children recently warned that with the complex humanitarian crisis in Lebanon worsening by the day, “time is running out” for Lebanon's children (4).

In this Perspective, we provide a brief overview of Lebanon's current complex crises and background context. Drawing largely on currently available rapid response operational reports from humanitarian agencies, we examine the impacts of this evolving situation on youth (children and adolescents up to 18 years of age) and propose a suite of recommendations to mitigate the effects.

## Background

Lebanon has a tumultuous contemporary history, stained with violent episodes at the hands of both local and foreign forces that have scarred the population psyche and yielded deep sectarian divisions and a corrupt, exploitative sectarian political landscape. The Lebanese civil war (1975–1990) resulted in up to 150,000 deaths, left over 100,000 severely injured and displaced up to two-thirds of the Lebanese population (5, 6). The Israeli invasion of Lebanon in 1982, and the 2006 Israeli war on Lebanon resulted in the deaths of over 17,000 and 1,109 people, respectively, predominantly civilians, and the destruction of critical infrastructures (6, 7). Syria maintained a strong presence in Lebanon from 1976 until its withdrawal following mounting pressure in 2005. Explosions and assassinations followed the murder of former Prime Minister Rafik Hariri in 2005; since then, ongoing political unrest continues to compound instability (8).

These events and Lebanon's political history have had major consequences on the Lebanese population (9–11) and are not divorced from the current crises. Vested regional interests today continue to yield considerable influence over internal Lebanese affairs. Many warlords directly responsible for atrocities committed during the civil war now comprise Lebanon's dynastic political establishment and the roots of many major political parties trace back directly to militias and military families from the civil war era (12). From these origins, the political establishment is rife with corruption. Nepotism and clientelism are widespread, including sectarian factions and political families typically providing support and wastas (“favours”) of anything from bread to basic services to jobs, in exchange for votes and allegiances (13, 14). Lebanon ranks 149th of 180 countries on the 2020 Index of Perceived Corruption within the public sector, with its ranking plummeting over recent years (15). According to Transparency International, 68% of citizens think that most or all government officials are involved in corruption, and 80% have little or no trust in the government (14). The state has long failed in its obligations to provide many services required to meet basic population needs. These failures and corrupt practises have had marked consequences on population health and well-being. These range from the impacts of the 2015–2021 garbage crisis (16), to cancer clusters associated with industrial dumping of pollutants by factories owned by or aligned with politicians (17–19), to state failure to provide reliable electricity supply generating heavy reliance on private, diesel-based electrical power generators that have increased household exposure to airborne carcinogens (20), to corruption exacerbating health and social inequalities and impacting access to healthcare (21). Corruption and a longstanding dysfunctional political establishment are also at the root of the current crises ravaging Lebanon and the failed government responses to address them.

### 2019 Protests and Economic Collapse

Ongoing governance failures, fiscal mismanagement and overt corrupt practises precipitated widespread protests across the country in October 2019 demanding political reform. The Lebanese economy has since been in freefall (22). The Lebanese pound (LBP), pegged since 1997 at 1507 LBP to 1 USD, devalued rapidly, losing up to 85% of its value. United-States-imposed sanctions and the smuggling of foreign currency across the border into Syria further exacerbated shortages of circulating USD (23). Banks imposed extreme capital control measures, such that people were unable to access their own USD savings (22). Curtailed imports due to foreign currency shortages caused massive food price inflation and rising food insecurity. A new humanitarian landscape emerged, with a large proportion of the Lebanese population joining already vulnerable refugee and marginalised groups requiring aid. Self-harm and suicides due to economic distress were reported in the media (24), and in April 2020 a government minister conceded that 70–75% of the population was in need of financial aid (25). A World Food Programme survey conducted in April–May 2020 reported that 50% of Lebanese respondents, 63% of Palestinians and 75% of Syrian refugees were worried that they did not have enough to eat in the preceding month; 44% of Syrians and one in five Lebanese and Palestinians reported only eating one meal over the previous day (26). In July 2020, Save the Children reported that 910,000 people in the Greater Beirut area, including 564,000 children, did not have enough money to cover the costs of basics, including sufficient food (27). Experts warned of imminent overt famine (28) and aid organisations warned they would start seeing children dying of hunger by the end of the year (27). Facing potential bankruptcy, in the midst of the pandemic many hospitals laid off staff and closed wards; deteriorating conditions also precipitated mass health worker emigration (29). Under-resourced and shaped by the impacts of multiple shocks since the civil war, Lebanon's public health system has further been deeply compromised by the current challenges (30). Whilst Lebanon has experienced numerous insults in the past, the compounded impact of several concurrent crises has been unprecedented (31).

### Beirut Blast

The Beirut Port blast on 4 August 2020 occurred due to the explosion of up to 2,750 tonnes of highly explosive ammonium nitrate that was illegally stored at the port, and which senior government figures had reportedly known of for years (32). The largest non-nuclear explosion in the 21st century (33), the blast left over 200 dead and around 6,500 injured, and it severely damaged around 40% of Beirut, crippled businesses and destroyed lives and livelihoods. With extensive damage to health infrastructures, the already stretched health system and its health workers were overwhelmed (34). In the absence of state support or a coordinated government response, the Lebanese population were left to drive clean-up efforts themselves—including many young people taking to the streets with brooms and dustpans to remove debris (35)—and left grappling with the psychological impacts of yet another crisis (36). Psychologists reported that the explosion triggered memories of the war among older Lebanese, many of whom had never dealt with their traumas and so did not know how to help their children following the blast (37). Humanitarian needs have since increased across the country, including in areas not directly affected by the explosion but experiencing the compounding impacts of the economic collapse, COVID-19 and port disruption (38–40). Needs have also soared among already vulnerable groups, including Syrian refugee households, nine out of ten of which reportedly now live in extreme poverty (41), and other foreign nationals (42).

### COVID-19

Following the first documented COVID-19 case on 21 February 2020, Lebanon implemented early and aggressive strategies that, until mid-2020, generally succeeded in containing COVID-19 spread (43). With social distancing and other COVID-19 containment efforts widely abandoned in the immediate aftermath of the port blast, during the massive clean-up efforts, and in the context of resumption of large-scale anti-corruption protests, COVID-19 cases soared. Lebanon imposed several short partial lockdowns in late 2020 which were poorly enforced, had poor compliance and met with strong resistance from businesses and lobby groups. With no social security benefits or income support, informal workers, who comprise an estimated 55% of the overall workforce and 95% of the non-Lebanese workforce, were unable to earn a wage, causing unprecedented hardships (44). In a bid to boost the economy and encourage expatriate return and spending over the end of year holiday period, Lebanon eased restrictions and opened up on 17 December 2020, several days before a nationwide lockdown was due to end (45). Early 2021 consequently saw a massive resurgence in COVID-19 case numbers, with Lebanon reporting at the time the Eastern Mediterranean region's highest daily case numbers per million population and record daily death tolls. PCR test costs and accessibility reportedly generated significant social disparities in test uptake, with official case statistics believed not to be reflective of the true disease burden and distribution (46). A strict 6-week total lockdown, including a national 24-h curfew, was imposed in an effort to control the outbreak (47). COVID-19 vaccination commenced on 14 February 2021, with first vaccines administered to priority groups, including healthcare workers and the elderly (48). Despite close coordination between the Lebanese Ministry of Public Health and WHO-Lebanon to centralise management of the COVID-19 vaccination program (49), corruption has also plagued the vaccine rollout, with reports of politicians securing and unofficially importing vaccines into the country for distribution to their loyalists and sectarian constituencies (50).

## The Current Situation

The current situation is widely described as the worst economic crisis in Lebanon's turbulent modern history. The World Bank has recently reported that the economic and financial collapse is among the most severe globally since the mid-19th century (51). The political stalemate and, as at the time of writing, ongoing failure to form a government has driven a sharp fall in the currency, which hit record lows in July 2021, unofficially trading at up to 24,000 LBP. The World Bank has warned that poverty in Lebanon will continue to worsen, likely surpassing half of the population by mid-2021, and that the country faces an arduous and prolonged recession (52). Many report that the current situation is worse than experiences during Lebanon's 15-year civil war. Food and medication shortages are now widespread. Many subsidised goods, including medications, are reportedly being smuggled out of Lebanon, further exacerbating supply-chain shortages (53). The oil and gas sector is nearing collapse (54). Petrol is scarce and the black market price soaring, with strict rations imposed, queues for fuel stretching for kilometres and transportation grinding to a halt. Imminent lifting of subsidies on essential items, combined with ongoing currency devaluation, will see further diminishing of purchasing power. Lifting of subsidies on fuel has also sparked concerns that Lebanon will soon literally be left in the dark, with electricity supplies at risk. As at July 2021, fuel shortages have led to widespread power blackouts, with ongoing electricity cuts lasting up to 22 h per day affecting more than 90% of the country (55). The healthcare system has been ravaged, and further compromised by brain drain (56). One in five doctors are believed to have either already left the country or intend to do so (57). Oxygen to support patients on breathing aid was in short supply in hospitals (58), and tonnes of oxygen had to be shipped in from Syria (59). Social tensions have soared (60) and violence and petty crimes have increased (61). Mental health professionals describe a collective psychological trauma (62).

Lebanon is in the throes of an overt humanitarian emergency, the impacts of which will be felt for generations. Children are among those particularly vulnerable. Due to sensitivities around sectarianism and population demographics, Lebanon has not conducted a national census since 1932. The World Bank estimates that of Lebanon's population (~6.85 million), 1.75 million are children aged 0–14 years (63). Hosting the largest number of refugees per capita, UNHCR estimates that Lebanon houses around 488,000 school-aged (3–18 years old) Syrian refugee children (64), around 121,380 Palestinian children (0–17 years old), and more than 5,000 Iraqi children (65), in addition to children of other nationalities, such as Sudanese and Ethiopian (66). Lebanese, refugee and migrant children alike have been impacted by the recent crises in the country.

## The Impact on Children of Lebanon's Complex Crises

### Schooling

Even prior to the Beirut blast, which left at least 183 schools damaged and is estimated to have affected access to learning and education support for more than 85,000 children and youth (3, 67), there were reports that 1,600 schools would close due to the economic crisis (68) and many children had been withdrawn from schools due to economic hardship (41, 69). These difficulties have soared across the country since the blast – a September 2020 survey of Lebanese and Syrian households from Mount Lebanon, Tripoli and Akkar districts, all a considerable distance from the blast site, reported that only 18% of households with children aged between 5 and 18 years could still afford schooling, including those whose children had attended public schools (39).

COVID-19 has further impacted educational access and uptake, with school closures in March 2020 estimated to have interrupted the education of more than 1.2 million children enrolled in private, public and UNRWA schools (67, 69) and ~30,000 children and youth enrolled in non-formal education (69). A remote online learning strategy was implemented by the Ministry of Education and Higher Education for all private and public schools (67). It is estimated that in 2019–20, children received a maximum of 11–18 weeks of schooling delivered by any modality (of 31–33 weeks in a traditional school year), and this figure is lower for Syrian children (70). Remote learning participation rates have varied considerably across governates, grade levels and student populations (67), mirrored by inequities in access to internet, computer facilities and electricity (41). In a survey of 137 Lebanese, Syrian and Palestinian children and youth between the ages of 12 and 24 years, a large proportion reported difficulties with distance-learning modalities, including up to 80% of female students (71). In a child-focussed survey of 1,244 households, around 25% of families reported being unable to afford online learning tools, including computer access and internet (3). Basic infrastructure and amenities shortages, including unreliable electricity supply and poor or limited internet access, are placing vulnerable children at further educational disadvantage (70).

It is estimated that at least one in four children in Beirut are now at risk of dropping out of school (72) and 40% of school-aged Syrian refugee children are not enrolled in any type of learning (69). It is reported that as at March 2021, 15% of 1,244 households surveyed by UNICEF had stopped their children's education (3). Access to education is further threatened by the imminent lifting of subsidies, with many children likely to either be transferred from private to public schools and others likely to forgo education completely as spending on food is prioritised over schooling, and as others are forced into child labour to support household incomes (73).

### Child Labour

Increasing rates of child labour have been reported since October 2019, including an increase in the number of children identified as being the sole breadwinners in their households (74). In the impoverished Bekaa region, nearly 70% of working household members are children aged between 4 and 18 years. Food insecurity is a main trigger for sending children to work (74), with increasing numbers of children being forced into income-generating activities due to escalating economic distress (69, 75, 76).

Children engaged in work and labour spend most of their time on the streets, where they are at high risk of exposure to exploitation (69). In a survey of 648 Lebanese and Syrian refugees in Bekaa, Beirut, North and South Lebanon, 18% of families with a working child report that the child works seven days a week, with no days off for rest or play (74). Notably, despite increasing rates of child labour, this has not alleviated food security pressures on families.

### Child Marriage

Rates of child marriage are reportedly increasing in Lebanon, particularly among Syrian refugee girls, to reduce the financial burden on families (3, 77, 78). Prior to the current crises, the prevalence of child marriage in Lebanon varied significantly by nationality (79), but across all groups the practise is becoming increasingly common as financial strains soar (74). Six months on from the Beirut blast, aid agencies warned that half a million children were at risk of child marriage or being forced into child labour due to the economic situation (80).

### Health and Nutrition

The direct health impacts of Lebanon's multiple crises on children are pronounced, and include an increased number of children aged under 5 years with acute malnutrition (69). UNICEF report that in March 2021, 30% of families in 1,244 households surveyed had at least one child who skipped a meal or went to bed hungry, and 77% of all households (99% of Syrian households) indicated that they did not have enough food or enough money to purchase food (3). Access to safe water is increasingly an issue, with 20% of households reporting insufficient drinking water (3). There have also been marked reductions in uptake and coverage of routine child immunisations, with a 43% decrease reported in private clinics (67), a 31% decrease at the national level, and 77% of paediatricians reporting a decrease in routine immunisation between October 2019 and April 2020 compared with the same period in the preceding year (81). UNICEF report that 30% of children are not receiving the primary health care they require, and 76% of households are impacted by soaring medication prices (3). “Period poverty” is also increasing with two-thirds of adolescent girls having no means of purchasing sanitary products, and many resorting to other measures to manage their periods, including newspaper, old rags, baby diapers or not leaving the house (82).

The health impacts of the Beirut blast on children are profound: more than 1,000 children were injured, 100,000 had their homes either completely or partially destroyed (83), and children from areas both near and far from the blast epicentre are experiencing psychological impacts related to trauma from the blast (67, 84, 85).

Physical and mental health problems are projected to continue to increase among children in Lebanon (86). Refugee and vulnerable children experience additional and particular psychosocial concerns, including, among Syrian refugee boys, a fear of violence and being targeted by the Lebanese authorities and surrounding communities (87) and fear about their future in Lebanon given the political and economic breakdown (87). Among working refugee boys, stress of being the sole breadwinners and fears that the economic situation may force them to engage in illegal activities such as drug dealing and cultivation are described (87). Negative coping mechanisms pose further health risks, with reports of alcohol and drug misuse among children of all nationalities in Lebanon, adopted as strategies to relieve the pressures of the pandemic and the economic crisis (87).

### Gender-Based Violence

An increase in violence against children and gender-based violence have been reported (67). Child poverty has increased considerably due to the multiple crises (67) and violence against women and girls is reported to have increased (69). Gender inequalities and discrimination have been disproportionately experienced by the poor and most vulnerable amongst the Lebanese, and Syrian and Palestinian refugees (88). Girls and women are at an increased risk of physical and sexual violence and exploitation (88). Pooled data from multiple service providers in Lebanon also demonstrate increases in gender-based violence and increases in reports of online bullying and attacks (89).

## Discussion

Lebanon was previously making modest progress towards achieving a number of the child-related sustainable development goals (90), but the current multifaceted crisis is reversing developmental gains. The adverse impacts on Lebanon's children span a range of domains and threaten every aspect of child well-being in both the immediate and longer terms. Exposure to childhood illness, malnutrition and injury can have implications across the lifespan. Increasing rates of school drop out, child marriage and forced labour condemn children to a life of increased vulnerability (69). Child marriage, a blatant violation of human rights, is associated with a range of adverse health and social outcomes (91). Child labour exposes children to a range of adverse health consequences, many of which may be experienced well into adulthood (92), and sets children on a trajectory to a lifetime of unskilled work and low earning capacity.

There have been numerous initiatives to provide immediate support and services in Lebanon, driven largely by local civil society, the local and international humanitarian sector and with support from the diaspora. For example, non-governmental organisations have established new mental health centres and launched awareness campaigns to encourage individuals to reach out through the national suicide prevention and mental health support call centres (93). Individuals and civil society organisations have actively driven assistance efforts, including the distribution of food aid, medicines, cash and clothing, opening kitchens and supermarkets to serve the most vulnerable and using land donated by wealthy individuals to grow and distribute produce to communities in need (94). Grassroots initiatives established after the blast, such as Khaddit Beirut, have collected donations and laptops for use by under-privileged students in public schools (95). The diaspora has also mobilised, including injecting millions of dollars into relief efforts, establishing initiatives such as the Beirut Emergency Fund 2020, and procuring and donating medical supplies and other emergency equipment (96). The Ministry of Public Health has also developed systemwide population-level approaches to address mental health issues (97), including the National Mental Health and Psychosocial Support Response to Beirut Explosion and the National Mental Health Response to COVID-19 programs (98). Immediate humanitarian assistance and search and rescue support following the blast was also provided as foreign aid. Despite such efforts, the lived experience of Lebanon's children remains increasingly dire.

The current humanitarian crisis is robbing Lebanon's youth of the most basic rights of the child to an adequate standard of living, education, health, protection and a life of dignity – rights that are firmly embedded in international agreements and ratified United Nations conventions (99). Although much needed, the rapidly deteriorating situation and increasing needs mean that local and international humanitarian agencies' efforts have not been sufficient to ensure that all vulnerable groups are reached. In a setting where the population is increasingly desperate, and its allegiances cheaper for corrupt political actors to buy, it is imperative that all in need can access required aid, regardless of political affiliation or creed. Addressing the root cause of Lebanon's crisis requires radical political reform, transparency, and accountability.

With the situation rapidly spiralling, there is an urgent moral and ethical imperative to move quickly to avert and mitigate the effects of the crisis engulfing Lebanon and putting an entire generation at risk. Those in Lebanon and in the diaspora may play crucial roles in mitigating the effects of the crisis. We urge the following.

### A Strengthened, Needs-Based and Locally Led Humanitarian Response

- An immediate, co-ordinated and equitable, needs-based social assistance package to prevent hunger and food insecurity. This intervention will reduce forced child engagement in income-generating activities and will increase school enrollment and participation. The Lebanese army that has gained the trust of both the Lebanese population and foreign actors and donors may be entrusted with managing the distribution of financial assistance.
- Development and delivery of educational packages through radio programs and televised educational modules, to address issues of poor/no internet and device access during periods of school closure. The Ministry of Education, in collaboration with international agencies, could develop these programs, with funding supplemented through private donations, including from the Lebanese diaspora. When the COVID situation allows, staged return to schools should be facilitated to support well-being and social connectedness (100).
- Economic reform to subsidise community-based healthcare services delivered at no out-of-pocket cost for those who cannot afford to pay. Recommending universal health coverage is unrealistic at this time due to the severe economic collapse in the country, but in the medium to longer term this must be addressed: the COVID-19 pandemic emphasised the necessity of healthcare access to all segments of society.
- An open, national dialogue about mental health to overcome taboos in a culture where mental health issues remain largely stigmatised and, in parallel, a ramping up of support services, including provision of free youth counselling services. School campaigns, social media and mass media messaging to normalise discussions about mental health and raise awareness about support avenues are also essential. Additionally, any rebuilding roadmap should include consideration of the importance of urban green spaces to mental health.
- An equitable, transparent humanitarian response. Delivery of aid and access to essential services must not be fragmented along sectarian lines. Lebanon's political elite and their sectarian factions' attempts to buy the allegiances of an increasingly desperate population through provision of assistance only to those who pledge their support can only be stopped through political reform.
- Strengthened support for a localised humanitarian response, with strict due diligence criteria. We echo calls that donors, international insitutions and UN agencies priortise partnering with vetted local and national humanitarian actors and sustainably build capacity, and strictly do not engage with or fund any entity with a history of corruption or allegiance to foreign powers with political agendas (94).

### Building the Evidence Base to Inform Action and Drive Accountability

- Improved data collection to inform targeted action. Paucity of and limited access to data on school enrolments, drop outs, attendance and participation are reportedly limiting development of educational interventions (70). Across all sectors, granular data are required to tailor interventions, inform funding allocations and ensure an equitable aid response is delivered to those most in need.
- Development of a locally led, locally coordinated research agenda that reflects local priorities and needs. The situation in Lebanon is rapidly evolving, and given the recency of events, to date there is limited published research documenting the humanitarian and population health impacts. Much rapid response operational research has been conducted and reported by aid agencies, but there is an urgent need for rigorous research and longitudinal follow-up studies on the impacts of these crises on children, to build the evidence base, inform action and drive accountability.

### Creating Local Opportunities, Supporting Local Initiatives and Mobilising the Lebanese Diaspora

- Create and support initiatives that generate employment opportunities for young people in Lebanon, including through online platforms that advertise roles with local and international organisations that are earmarked for job seekers in Lebanon, and through supporting local Lebanese businesses, many of which sell products online and distribute globally. The creation of these employment opportunities will help prevent child labour that has been increasing due to poverty.
- Ongoing provision of remittances from the Lebanese diaspora, and active diaspora support and engagement in rebuilding, restoring and supporting Lebanon's human capacity and its ravaged infrastructure. Skilled diaspora from diverse professions and disciplines can support local peers and contribute, from on-the-ground service, to remote capacity-building efforts, to mentoring a new generation who will help revive and rebuild Lebanon.

### Political Reform

- Urgent and radical political reform. The root causes of this crisis and its mismanagement are political. Fundamental and sweeping reforms are needed, and calls for the condemnation of sectarian politics, corrupt governance and a failed, nepotistic political establishment must be heeded.
- Accountability. Pressure from the local population is required to ensure implementation of anti-corruption legislation such as Law No. 189/2020 (Financial Disclosure and the Punishment of Illicit Enrichment Law), which removed ministerial immunity on corruption-related crimes, and Law No. 175/2020 (Combatting Corruption in the Public Sector) (101).

## Conclusion

The current crises engulfing Lebanon present a range of health, economic and social challenges that threaten the well-being of a generation of children. Whilst the whole world is reeling from the COVID-19 pandemic, and several other countries, such as Greece and Venezuela, have also experienced massive economic collapses, Lebanon's current situation is further complicated by the compounding effects of the Beirut blast and the backdrop of the Syrian refugee crisis, the United-States-imposed sanctions, the influence of regional and international actors and their local proxies, entrenched corruption, culture of nepotism and entitlement among the political dynasties, dysfunctional power-sharing, deep-seated sectarian divides - and a population that carries the scars of Lebanon's history of conflict and its ongoing insults. The root causes of this complex crisis, its protracted nature and ongoing mismanagement are deeply rooted in political corruption. The Lebanese people, and in particular the children of Lebanon, can be rescued only through rigourous political reform that demands merit-based and transparent governance and a political agenda that strengthens institutions and infrastructures, allows a life of dignity and protects the rights and well-being of all.

## Data Availability Statement

The original contributions presented in the study are included in the article/supplementary material, further inquiries can be directed to the corresponding author/s.

## Author Contributions

RH and MA: conceptualisation and drafting an early draft of the manuscript. DH, MA, and RH: literature review. RH, MA, DH, and AM: revising the final draft of the manuscript. All authors approved the final version of the manuscript.

## Conflict of Interest

The authors declare that the research was conducted in the absence of any commercial or financial relationships that could be construed as a potential conflict of interest.

## Publisher's Note

All claims expressed in this article are solely those of the authors and do not necessarily represent those of their affiliated organizations, or those of the publisher, the editors and the reviewers. Any product that may be evaluated in this article, or claim that may be made by its manufacturer, is not guaranteed or endorsed by the publisher.
